# Ecosystem functions including soil organic carbon, total nitrogen and available potassium are crucial for vegetation recovery

**DOI:** 10.1038/s41598-018-25875-x

**Published:** 2018-05-15

**Authors:** Kaiyang Qiu, Yingzhong Xie, Dongmei Xu, Richard Pott

**Affiliations:** 1 Institute of Geobotany, Leibniz Universität Hannover, 30167 Hannover, Germany; 2 Institute of Grassland Sciences, Ningxia University, Yinchuan, 750021 China

## Abstract

The effects of biodiversity on ecosystem functions have been extensively studied, but little is known about the effects of ecosystem functions on biodiversity. This knowledge is important for understanding biodiversity-ecosystem functioning relationships. Desertification reversal is a significant global challenge, but the factors that play key roles in this process remain unclear. Here, using data sampled from areas undergoing desertification reversal, we identify the dominant soil factors that play a role in vegetation recovery with ordinary least squares and structural equation modelling. We found that ecosystem functions related to the cycling of soil carbon (organic C, SOC), nitrogen (total N, TN), and potassium (available K, AK) had the most substantial effects on vegetation recovery. The effects of these ecosystem functions were simultaneously influenced by the soil clay, silt and coarse sand fractions and the soil water content. Our findings suggest that K plays a critical role in ecosystem functioning and is a limiting factor in desertification reversal. Our results provide a scientific basis for desertification reversal. Specifically, we found that plant biodiversity may be regulated by N, phosphorus (P) and K cycling. Collectively, biodiversity may respond to ecosystem functions, the conservation and enhancement of which can promote the recovery of vegetation.

## Introduction

Desertification is a global eco-environmental problem impacting 25% of the total terrestrial area^1,2^ and more than 250 million people worldwide^3^. Desertification was once considered to be an irreversible process^4^. However, studies have shown the recovery of vegetation in large areas of the Sahel from 1982 to 1999^5,6^ and a significant decrease in the area of the Sahara desert in the period between 1984 and 1994^7^. Moreover, Recent studies have demonstrated not only ongoing desertification^8^ but also the reversal of desertification in several regions of the world, such as North America^2^, Sub-Saharan Africa^9^, and regions of the Mediterranean^10^, suggesting the possibility of recovery from the equilibrium between desertification and its reversal.

Past studies involving alternative stable state theory support the idea that desertification is reversible^2,11^. Studies have documented that alternative stable states exist in both semi-arid grasslands and arid rangelands^11,12^. Recent studies summarized in^11^ suggested that desertified ecosystems may shift back to an alternative recovery state under certain favorable conditions, such as long term livestock removal. The mechanism of the transition from a desertified state to a reversed state has been demonstrated by new theoretical models which have illustrated that the absence of livestock gradually decreases soil compaction, resulting in higher soil water infiltration and availability. Thus, better conditions for the recovery of perennial grasses are provided, as soil water availability is a limiting factor for arid ecosystems^2,13,14^. These findings further suggest that drastic shifts can occur in desertified ecosystems, which can switch from the state of desertification to its reversal, assuming a sufficient perturbation to the state variables^15,16^. Several measures (as perturbations for the state of desertification), such as livestock removal, planting trees and grasses, and protecting areas susceptible to wind erosion (e.g. slopes), have been shown to be useful methods in promoting the transition of a desertified grassland to a recovery state. The combined effects of these measures may provide sufficient perturbation to the state variables and thus produce the reversal of desertification.

Although desertification has been shown to be reversible, there is still no established scientific definition of desertification reversal. Hence, we here define it as a process of desertified land returning to a less desertified state, including a series of changes in vegetation condition, soil properties and wind erosion. These changes may be due to natural or anthropogenic factors including climate change and human measures such as livestock removal. In this definition, vegetation condition includes plant productivity, biodiversity, species composition^9^, and a recovery of perennial grasses^2,13^. Focusing on desertification reversal is advantageous for understanding and combating further desertification. Vegetation recovery is seen as a key process in desertification reversal^14^. Key factors in soil may regulate and control the process and direction of vegetation recovery, which is a core issue in desertified ecosystems worldwide. In this study, we have therefore focused on the key soil factors for vegetation recovery, and our results have remarkable implications for global environmental policy.

As an important facet of desertification reversal, for more than 20 years biodiversity has frequently been reported as having a link with ecosystem functioning^17–19^. Most of these studies have focused on the effects of biodiversity on ecosystem functioning^20^. However, although evidence indicates that biodiversity also responds to ecosystem functions^17,21,22^, this feedback in biodiversity-ecosystem functioning (BEF) relationships is poorly understood. Moreover, biodiversity has not been considered with ecosystem functions related to plant production (plant cover and biomass)^21^ to evaluate vegetation recovery in desertification reversal. Plant cover is a significant factor in the structure and functioning of an ecosystem^23^, and plant biomass is highly related to plant productivity^24^. Plant biodiversity is an indicator of ecosystem functioning in drylands^23^ and is used as a measurement of vegetation recovery^25^. In combination, evaluations of plant cover, biomass, and biodiversity can therefore comprise a comprehensive assessment of vegetation recovery.

It is important to consider soil-vegetation relationships when addressing vegetation recovery. Previous studies have shown that soil degradation can promote changes in vegetation patterns, such as in plant community composition, during desertification^26,27^. In vegetation recovery, soil properties such as organic carbon and water content have also been determined to be the main factors driving plant distribution in dunes^28,29^. Soil factors play different roles in plant growth, development, and production and ecosystem functioning. However, the relative importance of ecosystem functions related to soil carbon and nutrient cycling and abiotic factors such as soil water condition for vegetation recovery is still unclear in desertification reversal. In particular, compared with carbon (C), nitrogen (N), and phosphorus (P), potassium (K) is more easily lost through soil erosion and runoff in drylands because it is not a component of the molecular structure of plants and is easily leached due to its small size^30^. However, K does plays an important role in plant growth and health^31^. Previous studies have found properties related to C, N, and P cycling that are key in ecosystem functioning^32,33^. Accordingly, the question of whether K cycling has a significance similar to that of C, N, and P cycling in desertified ecosystems has been raised. In order to address this and the relative importance of abiotic factors as well as ecosystem functions related to soil carbon and nutrient cycling, we propose the following hypotheses: ecosystem functions related to soil C, N, and P cycling are more significant than those related to K cycling for the vegetation recovery in desertification reversal; abiotic properties such as soil water content and soil texture also have significant effects on vegetation recovery but their effects are mediated by properties of the above carbon and nutrient cycling.

We investigated the effects of ecosystem functions on vegetation recovery that were related to soil C (organic C, SOC; catalase activity, CAT; and invertase activity, INV), N (total N, TN; available N, AN; urease activity, URE; and protease activity, PRO), P (total P, TP; available P, AP; and phosphatase activity, PHO), and K (total K, TK; and available K, AK) cycles and to primary abiotic factors (clay and silt fraction, CLS; very fine sand fraction, VFS; fine sand fraction, FS; coarse sand fraction, CS; soil bulk density, BD; water content, SW; pH; and electrical conductivity, EC) in areas that were undergoing desertification reversal in Mu Us Sandy Land, China. Vegetation survey and soil sampling plots were evenly assigned to five stages of desertification reversal. We analysed the above 20 soil properties as they are fundamental properties and are considered key for ecosystem functioning and desertification reversal^32,34^. We measured these soil properties and used the derived data as input data for a comprehensive analysis to test our hypotheses.

## Results

### Relationships between vegetation recovery and soil factors: Ordinary least squares (OLS) models

In desertification reversal, plant cover was significantly correlated with soil AK (p < 0.001), soil organic carbon (SOC) (p < 0.001), and the soil CS fraction (p < 0.01), which together explained 67% of the variation in plant cover (Table 1). Plant biomass was significantly associated with AK (p < 0.001), SOC (p < 0.01), TN (p < 0.05), PRO (p < 0.05), and phosphatase activity (PHO) (p < 0.05). These factors explained 65% of the variation in plant biomass. Plant species richness was significantly correlated with TN (p < 0.001), PHO (p < 0.01), and CLS (p < 0.05), which together explained 51% of the variation in plant species richness. Plant species diversity was associated significantly with soil AK (p < 0.01), SW content (p < 0.001), and PRO (p < 0.05) (Table 1). In particular, AK was significantly correlated with all four parameters of vegetation recovery except plant species richness. All four ordinary least squares (OLS) models were significant at p < 0.001.

**Table 1:** OLS models generated in the multiple regression analyses with single soil factors.

	Cover	Biomass	Richness	Diversity
*AK*				
B	49.22***	8.30***	—	0.81**
SEB	5.73	1.24		0.25
95.0% conf inter	37.83~60.60	5.84~10.77		0.31~1.30
Beta	**0.56**	**0.54**		**0.35**
*SOC*				
B	15.35***	1.82**	—	—
SEB	2.78	0.63		
95.0% conf inter	9.83~20.86	0.58~3.07		
Beta	**0.35**	**0.23**		
*CS*				
B	−3.01**	—	—	—
SEB	1.03			
95.0% conf inter	−5.07~−0.96			
Beta	−**0.19**			
*TN*				
B	—	2.24*	5.77***	—
SEB		0.99	1.12	
95.0% conf inter		0.26~4.22	3.54~7.99	
Beta		**0.24**	**0.74**	
*PRO*				
B	—	−0.58*	—	0.092*
SEB		0.26		0.047
95.0% conf inter		−1.10~−0.065		−0.001~0.19
Beta		−**0.20**		**0.21**
*PHO*				
B	—	1.45*	2.04**	—
SEB		0.70	0.68	
95.0% conf inter		0.056~2.85	0.68~3.40	
Beta		**0.18**	**0.30**	
*CLS*				
B	—	—	−1.68*	—
SEB			0.67	
95.0% conf inter			−3.02~−0.34	
Beta			**0.33**	
*SW*				
B	—	—	—	0.71***
SEB				0.21
95.0% conf inter				0.30~1.12
Beta				**0.30**
*Constant*				
B	−76.46	−10.79	9.53	−1.08
SEB	13.68	2.96	1.80	0.46
95.0% conf inter	−103.63~−49.30	−16.67~−4.92	5.96~13.10	−1.99~−0.16
Adjusted R^2^	0.67	0.65	0.51	0.31
P_f_	0.00	0.00	0.00	0.00
SEE	11.05	2.00	2.04	0.42
n	95	94	96	96

Notes: Each column represents an OLS model using a vegetation recovery parameter as a dependent variable and soil factors as independent variables. The standardized coefficients are in bold. ***P_t_ < 0.001; **P_t_ < 0.01; *P_t_ < 0.05. P_t_, p value of the t-test for an individual regression coefficient. B, un-standardized coefficient; SEB, standard error of B; 95.0% conf inter, 95.0% confidence interval for B (lower bound~upper bound); Beta, standardized coefficient; P_f_, p value of the F-test for the overall significance of the model; SEE, standard error of the estimate; n, number of cases.

The OLS models evaluating the relationships between vegetation recovery and soil C, N, P, and K cycles showed that all four parameters of vegetation recovery except for plant species diversity were significantly correlated with K cycling (Table 2), which highlights the critical role of K cycling. In addition, the vegetation recovery parameters linked to plant growth and production (plant cover and biomass) were significantly associated with C cycling, whereas the plant biodiversity parameters (plant species richness and diversity) were significantly associated with N cycling. In desertification reversal, 55% and 45% of the variation in plant cover and biomass was explained by C and K cycling, respectively. In total, 43% of the variation in plant species richness was explained by K, N, and P cycling. N cycling explained 21% of the variation in plant species diversity, and C, K, and N cycling explained 56% of the variation in the general index of vegetation recovery (REC). Soil C, N, and K cycling showed relationships of similar strength with REC (Table 2). All the OLS models were modified to eliminate potential autocorrelations among residuals (Tables 3 and 4). Soil AK, SOC, and TN still showed significant relationships with most of the vegetation recovery parameters with which they were correlated in the models prior to modification for autocorrelation (Table 3) although C cycling no longer exhibited significant associations in the modified models (Table 4). The K and N cycles were still significantly associated with all the vegetation recovery parameters that they were associated with before the model modification.

**Table 2:** OLS models generated in the multiple regression analyses between vegetation recovery and soil C, N, P and K cycles.

	Cover	Biomass	Richness	Diversity	REC
*C cycling*					
B	0.66***	0.58***	—	—	0.32**
SEB	0.090	0.095			0.12
95.0% conf inter	0.48~0.84	0.39~0.77			0.084~0.55
*K cycling*					
B	0.52***	0.40***	0.25*	—	0.29***
SEB	0.093	0.099	0.11		0.081
95.0% conf inter	0.33~0.70	0.21~0.60	0.026~0.47		0.13~0.45
*N cycling*					
B	—	—	0.38*	0.54***	0.30*
SEB			0.15	0.10	0.12
95.0% conf inter			0.087~0.67	0.33~0.74	0.066~0.52
*P cycling*					
B	—	—	0.33*	—	—
SEB			0.15		
95.0% conf inter			0.026~0.63		
*Constant*					
B	0.027	−0.030	0.001	0.000	0.001
SEB	0.069	0.072	0.077	0.090	0.055
95.0% conf inter	−0.11~0.16	−0.17~0.11	−0.15~0.15	−0.18~0.18	−0.11~0.11
Adjusted R^2^	0.55	0.45	0.43	0.21	0.56
P_f_	0.00	0.00	0.00	0.00	0.00
SEE	0.67	0.70	0.75	0.89	0.54
n	95	95	96	96	96

Notes: Each column represents an OLS model using one of the vegetation recovery parameters as a dependent variable and soil C, N, P and K cycles as independent variables. The coefficients of C, N and K cycles are presented, and significant values are in bold. ***P_t_ < 0.001; **P_t_ < 0.01; *P_t_ < 0.05. P_t_ denotes p value of the t-test for an individual regression coefficient. REC denotes the general index of vegetation recovery. The other abbreviations are as presented in Table 1.

**Table 3:** Revised OLS models of the multiple regression analyses between vegetation recovery and soil factors.

	Cover	Biomass	Richness	Diversity
*AK*				
B	39.80***	6.82***	—	0.76**
SEB	6.88	1.33		0.27
*SOC*				
B	10.21**	0.72	—	—
SEB	3.23	0.65		
*CS*				
B	−3.60**	—	—	—
SEB	1.17			
*TN*				
B	—	2.39*	5.12***	—
SEB		1.00	1.10	
*PRO*				
B	—	−0.40	—	0.10*
SEB		0.24		0.049
*PHO*				
B	—	1.04	1.84*	—
SEB		0.67	0.73	
*CLS*				
B	—	—	−1.07	—
SEB			0.68	
*SW*				
B	—	—	—	0.71**
SEB				0.22
*Constant*				
B	−47.49**	−5.94	8.96***	−0.99*
SEB	16.59	3.21	1.76	0.50
*AR (1)*				
B	0.46***	0.51***	0.30**	0.23*
SEB	0.10	0.098	0.10	0.10
*AR (4)*				
B	—	—	—	−0.22*
SEB				0.11
Adjusted R^2^	0.71	0.71	0.53	0.37
P_f_	0.00	0.00	0.00	0.00
SEE	10.35	1.82	1.98	0.41
n	94	93	95	92

Notes: Autocorrelation was eliminated using the generalized difference method with EViews version 8. AR (1) and AR (4) denote the estimated values of the coefficients for first- and fourth- order autocorrelation, respectively. ***P_t_ < 0.001; **P_t_ < 0.01; *P_t_ < 0.05. P_t_ denotes p value of the t-test for an individual regression coefficient. The other abbreviations are as presented in Table 1.

**Table 4:** Revised OLS models generated in the multiple regression analyses between vegetation recovery and soil C, N, P and K cycles.

	Cover	Biomass	Richness	Diversity	REC
*C cycling*					
B	0.18	0.069	—	—	−0.083
SEB	0.13	0.13			0.11
*K cycling*					
B	0.38***	0.30***	0.27*	—	0.29***
SEB	0.097	0.08	0.12		0.071
*N cycling*					
B	—	—	0.33*	0.49***	0.33**
SEB			0.15	0.13	0.10
*P cycling*					
B	—	—	0.43**	—	—
SEB			0.15		
*Constant*					
B	0.044	−0.0089	9.53	0.0071	0.036
SEB	0.19	0.16	1.80	0.13	0.16
*AR (1)*					
B	0.67***	0.63***	0.44***	0.32**	0.73***
SEB	0.082	0.089	0.095	0.10	0.075
Adjusted R^2^	0.64	0.61	0.53	0.28	0.72
P_f_	0.00	0.00	0.00	0.00	0.00
SEE	0.60	0.59	0.68	0.85	0.43
n	94	94	95	95	95

Notes: The autocorrelation was eliminated using the generalized difference method in EViews version 8. REC denotes the general index of vegetation recovery. AR (1) denotes the estimated values of the coefficients for first-order autocorrelation. ***P_t_ < 0.001; **P_t_ < 0.01; *P_t_ < 0.05. P_t_ denotes p value of the t-test for an individual regression coefficient. The other abbreviations are as presented in Table 1.

### Relationships between vegetation recovery and soil factors: individual effects

**Figure 1:**
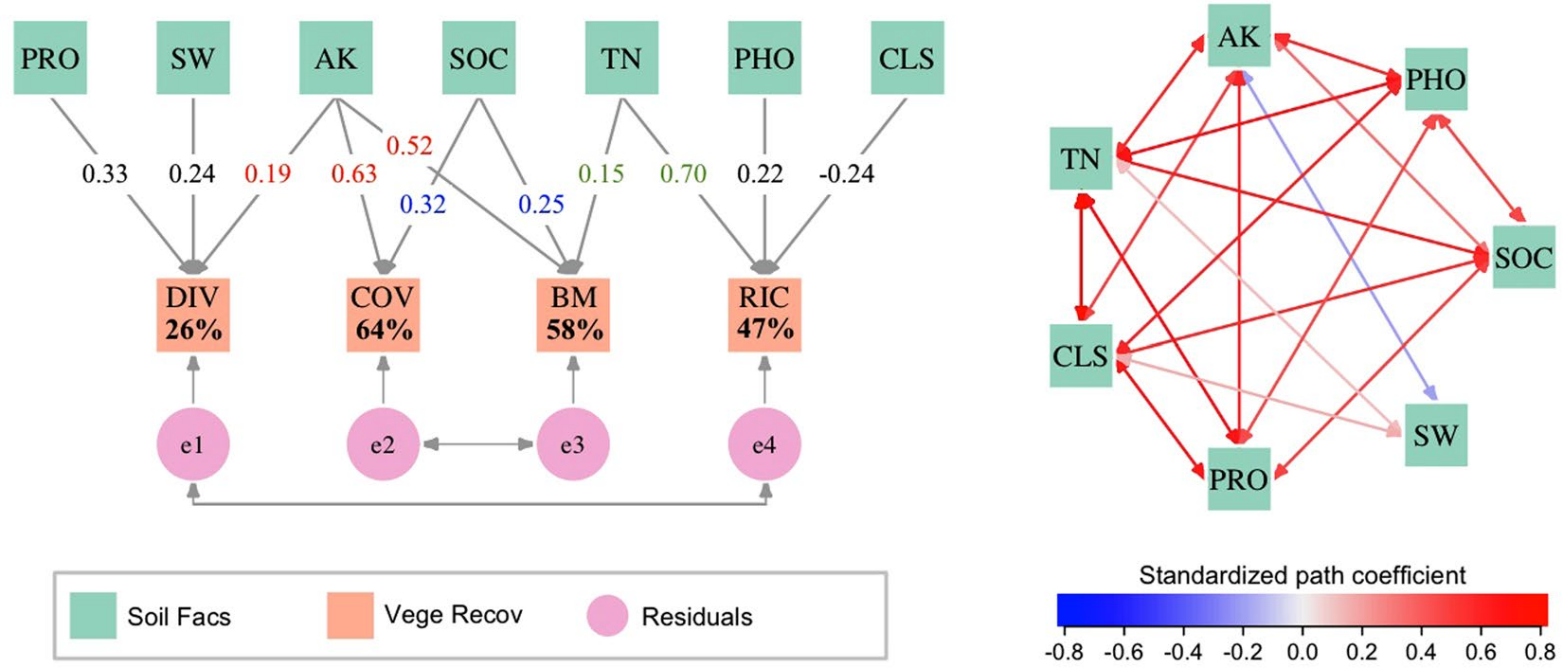
Structural equation model (SEM) without latent variables (SEM1) showing individual effects of soil factors on each parameter of vegetation recovery. Single-headed arrows show the effects of soil factors on vegetation recovery and the residuals of endogenous variables. Double-headed arrows indicate correlations between two variables. Numbers on arrows represent the standardized path coefficients, and a number under the corresponding variable is the percent of its variance explained by the predictors. Soil Facs, soil factors; Vege Recov, parameters of vegetation recovery. For other abbreviations, see the main text.

The structural equation model (SEM) without latent variables (SEM1) explained 64, 58, 47, and 26% of the variance in plant cover (COV), biomass (BM), species richness (RIC), and diversity (DIV), respectively (Fig. 1). Soil AK showed significant effects on all four parameters of vegetation recovery except RIC, and COV and BM were mostly affected by AK, indicating that AK has an important role in vegetation recovery. Soil TN had significant effects on both BM and RIC, and soil PRO had a significant effect on DIV. These findings indicate the importance of ecosystem functions related to N cycling. Similarly, SOC had significant effects on both COV and BM (Fig. 1), suggesting the importance of C cycling as SOC is the main component of the soil carbon pool and is a prominent constituent of the global terrestrial carbon pool^35^. SEM1 also indicated intricate correlations among soil factors in the desertification reversal process. Soil AK, TN, and CLS were significantly correlated with all of the other included soil factors, and SOC, PRO, and PHO were significantly correlated with all of the other soil factors except water content (Fig. 1).

### Relationships between vegetation recovery and soil factors: general effects

**Figure 2:**
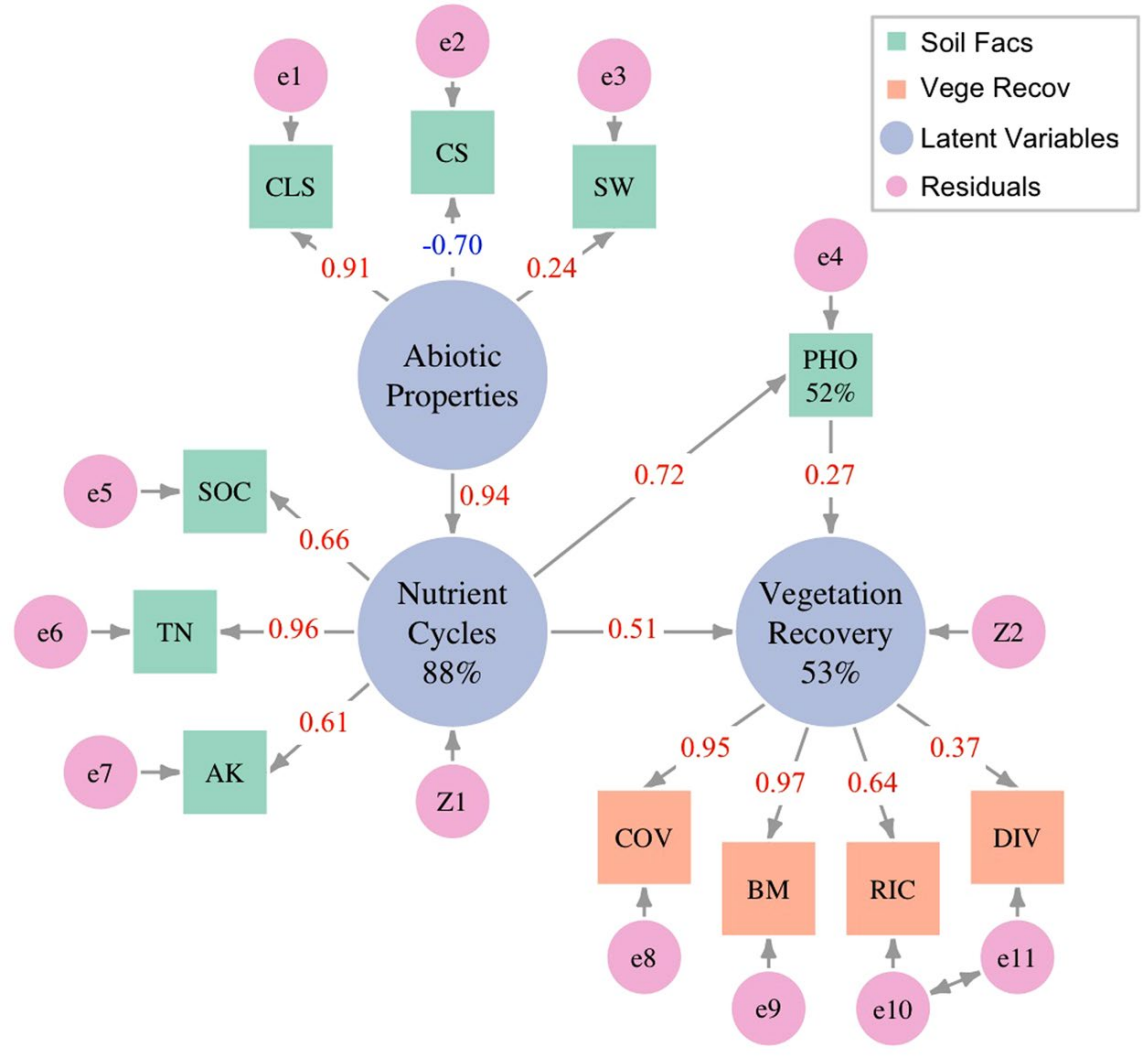
Structural equation model (SEM) with latent variables (SEM2) revealing the overall effects of different groups of soil factors on the whole group of vegetation recovery factors. Single-headed arrows in the centre of the figure show the effects of groups of soil factors and PHO on vegetation recovery. The other single-headed arrows show residuals and the formation of latent variables. Double-headed arrows indicate the correlation between two residuals. The term “abiotic properties” represents soil abiotic properties (SAP), and “nutrient cycles” denotes ecosystem functions related to soil carbon and nutrient cycling.

In the SEM with latent variables (SEM2), only ecosystem functions related to soil carbon and nutrient cycling (NC) and PHO showed significant effects on vegetation recovery (P < 0.05) (Fig. 2). In total, 53% of the variance in vegetation recovery was directly and indirectly (mediated by PHO) accounted for by NC. Soil abiotic properties (SAP) showed a substantial indirect effect on vegetation recovery through the mediation of NC and PHO (Fig. 2) with a standardized coefficient of 0.66, which is close to that of the total effect of NC (0.70). These results verify the predominant effects of AK, SOC, and TN on vegetation recovery but also indicate the key roles of CLS,CS, and SW content. However, the effects of CLS, CS, and SW were indirect and mediated by NC and PHO.

## Discussion

Soil functions are a vital part of living systems^36^ and provide support and resources for vegetation development. In this study, we analysed the relationships between soil variables and vegetation recovery to identify the most significant soil factors for vegetation recovery in desertification reversal and to provide useful information for land management planning in desertified regions. We analysed 20 soil properties that comprehensively represent soil conditions from all aspects and can present an overall state of the soil. Using such a broad range of soil properties is useful in discovering the “true” key soil factors for vegetation recovery in desertified ecosystems. Conducting analyses with only a portion of these soil variables may neglect some important factors, which in turn would lead to a severe shortcoming in the models. We therefore analysed these 20 soil variables for each soil sample.

### Critical soil factors for vegetation recovery

The critical effects of soil AK on vegetation recovery found in this study imply that AK may regulate plant growth and species distribution in desertification reversal as the latter is linearly correlated to species diversity at the plot scale^37^. Our results also imply that K, which is frequently lost by runoff and foliar leaching^30^, is a limiting factor for vegetation recovery. Sandy soils are particularly prone to K deficiency^38^. A soil K deficiency may weaken the ability of plants to resist drought stress in arid and semi-arid regions^31^. Numerous studies have shown that K deficiency can limit plant growth and production^30,39^, which is in accordance with our results. Our study also suggests that soil K deficiency plays a role in limiting plant biodiversity, which also implies that K contributes to increasing plant biodiversity in desertification reversal. Soil properties linked to C, N, and P cycling are considered key in ecosystem functioning^32^, while K cycling is unfortunately neglected in the literature^30^. The critical roles of AK found in our study suggest that K cycling is also a critical determinant for ecosystem functioning, which is the joint effect of all processes maintaining an ecosystem, in desertification reversal^17^.

The key roles of C and N cycling in ecosystem functioning found in our study are consistent with those of previous studies in dryland and wetland ecosystems^32,33^. Specifically, our OLS models showed that variations in plant production can be explained by K and C cycling, indicating that plant growth may be tied mainly to K and C cycling. Ecosystem functions related to these two cycles may be critical for vegetation recovery, including plant growth and production recovery, in the process of desertification reversal. Variations in plant biodiversity could be explained by K, N, and P cycling, suggesting that ecosystem functions related to K, N, and P cycling might be crucial for the recovery of plant biodiversity. These results further indicate that ecosystem functions linked to C cycling contribute mainly to plant growth and production and that the functions related to N cycling contribute mainly to plant biodiversity. Related to C cycling, SOC was found to be a critical factor for plant growth and production in desertified ecosystems, and this finding was in accordance with previous studies in other ecosystems^40^. SOC accounts for approximately 67% of the total terrestrial carbon pool^41^ and is a key property related to global carbon cycling^42^. SOC affects plant production and acts as a critical control of soil fertility and agricultural production^40^.

Furthermore, the substantial indirect effects of CLS, CS, and SW content suggest a mechanism in which soil factors impact vegetation recovery. CLS, CS, and SW regulate ecosystem functions linked to cycles of soil C (SOC), N (TN), and K (AK), which directly affect vegetation recovery. Water is the basis for plant metabolism because all chemical factors that impact soil metabolism, including C, N, P, K, and their compounds, are dissolved in or transported by water. Water deficiency is also one of the main factors in desertification. Our results highlight the indirect effects of SW on vegetation recovery. Our SEM1 showed intricate correlations between soil factors in desertification reversal, suggesting that soil factors impact vegetation recovery through a complex process in which the soil factors interact with each other in intricate ways. Collectively, the ecosystem functions related to soil C, N, and K cycling are critical for vegetation recovery in desertification reversal. These chemical factors not only exerted direct effects but also mediated the indirect effects of soil texture and water content on vegetation recovery. The predominant factors for vegetation recovery found in this study provide a scientific basis for desertification reversal and land management in arid and semi-arid regions.

### Implications for biodiversity-ecosystem functioning (BEF) relationships

BEF relationships have been a central issue in ecology during recent decades^22^. Past studies have mainly focused on the effects of biodiversity on ecosystem functioning^20^. We recognize these effects, which were demonstrated by extensive research. However, biodiversity also responds to ecosystem functions^17,21,22^. This response is significant for understanding BEF relationships because it provides an unbiased perspective of the interactions between both sides. How biodiversity responds to ecosystem functions has not been specifically examined. Our study documented this feedback by a series of comprehensive analyses with consideration of two facets of plant biodiversity, i.e., plant species richness and species diversity. We found that ecosystem functions related to soil N (TN) and P (PHO) cycling and CLS are critical for plant species richness and that ecosystem functions linked to K (AK) and N (PRO) cycling and SW content are especially significant for plant species diversity. Our results highlight the significance of soil TN and PRO for plant biodiversity in desertification reversal. TN represents the magnitude of the pool of N in the soil. Soil protease decomposes proteins and peptides into amino acids^43^, which is the first stage of soil nitrogen mineralization^44^. As both TN and PRO are important properties related to N cycling, our findings further suggest a critical role of N cycling in maintaining and increasing plant biodiversity during the reversal of desertification. Our study provides a new perspective on BEF relationships and contributes to this field by revealing the critical ecosystem functions of two facets of biodiversity. Moreover, our study advances this field by combining biodiversity with primary production parameters. This approach enables the comprehensive assessment of biodiversity rather than the assessment of biodiversity alone. Examining a combination of biodiversity and other plant and vegetation attributes increases the role of biodiversity and highlights its central status in ecology. BEF relationships need to be considered as part of more complex processes to disentangle ecological issues such as desertification reversal and climate change. Finally, our study is also significant for biodiversity conservation, which has been documented in several previous studies that dissected the ecological consequences of biodiversity loss and thus the effects of biodiversity on ecosystem functioning^45,46^. However, our study identified the ecosystem functions that are critical for biodiversity. This information benefits biodiversity conservation by informing scientific guidelines for improving land management.

## Conclusions

Using data sampled from 96 plots in five stages of desertification reversal, we analysed the relationships between vegetation recovery and soil factors by a series of comprehensive analyses with OLS and SEM. The results partially support our hypotheses. Contrary to our expectations, soil AK played critical roles in both plant production and plant biodiversity. These findings indicate that K is a limiting factor for vegetation recovery and is an important component of ecosystem functioning. AK, SOC, and TN are the most important soil factors in desertification reversal and mediate the indirect effects of soil texture and water content on vegetation recovery. Although soil factors interact with each other in intricate ways, plant growth and production may be regulated by soil C and K cycling, while plant biodiversity may be regulated by soil N, P, and K cycling. Ecosystem functions related to N cycling are critical in maintaining and increasing plant biodiversity. This study also confirmed that incorporating BEF into a more complex context, such as vegetation recovery, can broaden the applications of BEF to address ecological issues such as desertification reversal.

## Methods

### Description of study sites

The study sites were located in Yanchi County (106°30′E–107°47′E, 37°04′N–38°10′N), Southern Mu Us Sandy Land, China. This region has a temperate continental climate^47^ with an annual mean temperature of 8.1 °C and an annual mean precipitation of 300 mm^48^. Desertification expansion occurred in this area under the influence of a combination of climate conditions and grazing pressure starting in 1961, and these impacts were especially prominent between 1961 and 1989^48^. However, desertification reversal has been occurring in this region since 1989 due to ecological measures such as the Three North-Shelter Forest Program and Grain for Green Project and especially due to the release of grazing pressure since 2002. This reversal has taken place without obvious changes in climate conditions. The Three North-Shelter Forest Program began in 1979 and aims to reduce desertification by planting trees. Grain for Green is a Chinese national project in which farmers grow grasses and trees instead of crops on steep croplands and in return receive grain or cash from the government as compensation. This project aims to increase vegetation cover and prevent soil erosion^49^. A prohibition on grazing was implemented for the whole region in 2002, providing the opportunity for desertification to further reverse naturally. Livestock in the region was then raised in sheepfolds by farmers at their homes. Studies have shown that grazing exclusion can efficiently promote desertification reversal^50^. In our study region, the reversal of areas that were previously dunes is clearly related to the sustained decrease in bare sand area, increase in plant cover, and reduction in soil erosion after the implementation of the ecological measures over several decades.

### Experimental design and sampling

Fieldwork was carried out during July and August in both 2012 and 2013. Seven study sites 25–100 ha in size were selected that were formerly dunes. At each site, we selected four to five sub-sites at different stages of desertification reversal [desertification reversal stage (DRS) 1, DRS2, DRS3, DRS4, and DRS5] primarily according to the proportion of bare sand area to total ground area and secondarily based on the vegetation cover^48,51–53^. Furthermore, we also considered the intensity of wind erosion and aeolian activity as subsidiary parameters when we selected the sub-sites^52,54^. A higher stage represents a more advanced reversal of desertification with a lower proportion of bare sand area, higher vegetation cover, and weaker wind erosion and aeolian activity than the lower stages. For more details about the criteria for the different stages of desertification reversal see Supplementary Table S1. We set the five stages of desertification reversal to ensure that the data collected evenly represent all of the phases of desertification reversal. We recognized that a reversal of desertification involves not only the recovery of plant cover, but also changes in plant productivity, species composition, biodiversity, and soil properties. We set the criteria for the selection of sub-sites mainly based on the proportion of bare sand area and vegetation cover because these criteria normally correspond to other vegetation characteristics such as plant productivity and soil properties, e.g. where there is a low proportion of bare sand area and high vegetation cover, there is normally relatively high plant productivity and the conditions of the soil properties are relatively good. Moreover, at the beginning we were only able to select the sub-sites, and then quantify the rest of vegetation characteristics such as plant biomass and biodiversity, and soil properties.

Each sub-site was approximately 20 m × 20 m. Three plots with a core area of 5 m × 5 m were assigned randomly and homogeneously distributed at every sub-site. The data analysed in this study were collected from 96 plots. Soil and plant sampling and vegetation investigations were conducted in the plots. We set a 1 m × 1 m quadrat in the centre of each plot. The abundance of every plant species was recorded. The total plant cover and aboveground biomass were measured. All plant species were recorded.

Simultaneously, five soil columns with a diameter of 5 cm were taken from positions homogeneously distributed throughout the quadrat to a depth of 20 cm. All five soil columns from the same quadrat were then completely mixed as one sample. These samples were used for the analyses of soil texture (soil particle size distribution), soil chemical properties, and enzyme activities. All the samples were sampled once again for the analyses of SW content and BD. Samples for the analysis of soil BD were taken with a cutting ring. All the 20 soil properties were analysed in the laboratory.

### Statistical analysis

For further statistical analysis, plant DIV, SW content, and soil BD were first calculated with their respective formulas (see Supplementary Information S2). Plant RIC was the number of plant species recorded in the vegetation investigation.

#### Multiple regression modelling

Based on field and laboratory measurements, we first built OLS regression models with SPSS 22.0.0.0 (IBM Corporation, Armonk, New York, USA) to select soil predictors for the parameters of vegetation recovery. First, we tested the linear relationship between vegetation variables and each of the soil variables with Pearson correlation coefficients. The soil variables without a significant correlation with a vegetation variable were excluded from the multiple regression analysis of this vegetation variable. Before OLS modelling, the raw data for all variables were tested for normality and were then normalized by transformation if they were not normally distributed. For the data transformation and process of identifying outliers, see Supplementary Information S3. For the multiple regression modelling procedures, see Supplementary Information S4.

#### OLS models for vegetation recovery and soil C, N, P and K cycling

To evaluate the general relationships between vegetation recovery and soil C, N, P and K cycling, we generated OLS multiple regression models using the cycling indices, which were the averaged values of the Z scores for all the analysed variables of a cycle^32^. For instance, the index of P cycling is the average of the Z scores of TP, AP, and PHO. In addition, we also calculated the REC, which is the average of the Z scores of plant COV, BM, RIC, and DIV. For details on the tests for linear relationships, data normality, and outliers prior to the multiple regression analyses, see Supplementary Information S5. Prior to constructing the OLS models, we tested the assumptions for multiple regression analyses and found that our data fulfil all assumptions (see Supplementary Information S6).

#### Elimination of potential autocorrelation among residuals

Potential autocorrelation among the residuals might exist in a specific model produced in the multiple regression analysis in this study. Autocorrelation was tested with the Q-Statistics correlogram feature in EViews version 8 (IHS Global Inc., Irvine, California, USA). If first- or high-order autocorrelation existed among residuals, the model was modified with a generalized difference method to eliminate the autocorrelation using EViews version 8.

#### Structural equation modelling (SEM)

Based on the multiple regression analyses, we applied SEMs to test the effects of soil factors on vegetation recovery with AMOS 21.0.0 (IBM SPSS, AMOS Development Corporation, Meadville, Pennsylvania, USA). Compared to other statistical methods, SEM has several advantages, such as measuring multiple effects simultaneously, allowing for the development and assessment of complex relationships at the system level^55^, and avoiding false significant relationships between two variables caused by significant relationships between a third variable and both of these two variables^56^.

An SEM without latent variables (SEM1) was first employed to test the effects of soil factors on individual vegetation recovery parameters because SEM1 incorporates each soil property and individual aspects of vegetation recovery. Latent variables make it possible to distinguish the difference between concepts of interest and observations in an SEM and can also address the effects of measurement error^55^. An SEM with latent variables (SEM2) was then utilized to show the overall effect of a group of soil factors, e.g. functions related to soil carbon and NC, on the whole group of vegetation recovery factors. We employed two models, SEM1 and SEM2, because they provide insight into different aspects of soil factor-vegetation recovery relationships. While SEM1 focuses on the individual effect of one soil factor on a vegetation recovery parameter, SEM2 focuses on the overall effect of a soil factor group on the vegetation recovery parameter group. These group effects aid our understanding of the relationships between soil factors and vegetation recovery more than individual effects because a soil factor group represents a more complex and higher level concept than the individual factors. We are especially interested in the effect of soil factors on the whole vegetation recovery parameter group, which addresses vegetation recovery as one variable. Nevertheless, the individual effects also provide significant information as they show a clear picture of the role of each soil factor in each vegetation recovery parameter.

Both SEMs were estimated with the maximum likelihood method. For descriptions of model fit indices, outliers, and multivariate normality, see Supplementary Information S7. Figures 1 and 2 were drawn using R studio (Version 1.0.136, RStudio, Inc., Boston, Massachusetts, USA) with R version 3.1.2 (2014-10-31). For all abbreviations and their full forms, see Supplementary Table S5.

### Data availability

The datasets generated and/or analysed during the current study are available from the corresponding authors on reasonable request.

## Electronic supplementary material

Supplementary Information
