# Supplementary material for: Ecosystem functions including soil organic carbon, total nitrogen and available potassium are crucial for vegetation recovery
Source: Sci Rep. 2018 May 15;8:7607. doi: 10.1038/s41598-018-25875-x (PMC5954079; doi:10.1038/s41598-018-25875-x)
Supplement: Supplementary file 1 — Supplementary Information [file 41598_2018_25875_MOESM1_ESM.pdf]

Supplementary Information for

Ecosystem functions including soil organic carbon, total nitrogen and available potassium are crucial for vegetation recovery

Kaiyang Qiu[1,*], Yingzhong Xie[2,*], Dongmei Xu[2] & Richard Pott[1]

[1] Institute of Geobotany, Leibniz Universität Hannover, 30167 Hannover, Germany

[2] Institute of Grassland Sciences, Ningxia University, 750021 Yinchuan, China

*Corresponding authors. Email: qiu@geobotanik.uni-hannover.de; xieyz@nxu.edu.cn

Submitted to ***Scientific Reports***

Supplementary Information S1.

Supplementary Table S1 Criteria for determining different stages of desertification reversal

Stages of reversal	Bare sand proportion (%)	Vegetation cover (%)	Wind erosion	Aeolian activity
1	> 50	< 10	Very strong	Very common
2	30-50	10-30	Strong	Very common
3	10-30	30-40	Medium	Common
4	<10	40-50	Light	Rare
5	0	> 50	No	No

The sub-sites at different stages of desertification reversal were selected primarily according to the proportion of the bare sand area to the total ground area, and secondarily based on the vegetation cover. Additionally, we also considered the intensity of wind erosion and aeolian activity as subsidiary parameters. Stages of reversal: stages of desertification reversal; bare sand proportion: the proportion of the bare sand area to the total ground area.

Supplementary Information S2. Calculation of plant species diversity, soil water content, and soil bulk density

Plant species diversity (DIV) was calculated with the Shannon-Wiener index [1-4] (Eq. S1).

$$\mathrm{H'} = -\sum_{\mathrm{i}=1}^{S} P_i \ln P_i \qquad (1)$$

where H' represents the Shannon - Wiener index; S represents the number of plant species; P_i represents the proportion of the abundance of species i to the abundance of all species (Eq. S2):

$$P_i = \frac{n_i}{NI} \qquad (2)$$

where n_i is the the number of individuals of species i; NI is the total number of individuals of all plant species.

Soil water content was calculated with the following equation (Eq. S3):

$$SW = \frac{W - D}{D} \times 100\% \qquad (3)$$

where W is the wet weight of the soil sample; D is the dry weight (after at least 8 hours drying in the oven at 105 ℃ until the weight of the soil remained constant) of the soil sample.

Soil bulk density was calculated with the following equation (Eq. S4):

$$BD = \frac{W}{(1 + SW) \times V} \times 100\% \qquad (4)$$

where BD is the soil bulk density; W is the wet weight of the soil sample in the whole cutting ring; SW is the soil water content; V is the volume of the cutting ring.

References

1 Chawla, A. *et al.* Correlation of multispectral satellite data with plant species diversity vis-à-vis soil characteristics in a landscape of Western Himalayan Region, India. *Appl. Remote Sens. J.* **1**, 1-13 (2010).

2 Magurran, A. E. *Ecological Diversity and Its Management*. (Princeton Univ. Press, 1998).

3 Belsky, A. J. Effects of grazing, competition, disturbance and fire on species composition and diversity in grassland communities. *J. Veg. Sci.* **3**, 187-200 (1992).

4 Shannon, C. E. & Weaver, W. *The Mathematical Theory of Communication*. (University of Illinois Press, 1962).

Supplementary Information S3. Data transformation (for normal distribution) and outlier diagnosis before the multiple regression analyses between vegetation recovery and soil factors

The raw data of some variables were square-root transformed, including BM, PRO, VFS, AP, CS, SOC, AN, CAT; the raw data of other variables were log-transformed, including EC, TN, CLS, AK, SW, TP, URE, PHO, INV; while the raw data of TK was transformed by log (TK-10), that of pH was square-transformed. For all abbreviations for soil factors and their full forms see Supplementary Information S8. After transformation, the data of each variable was fully or at least approximately normally distributed. Data with a residual, from which the mean of all residuals in a variable was subtracted and a result of more than three times the standard deviation of all residuals was reached, were considered to be outliers and removed from the data set before the regression analysis. In the multiple regression analysis between COV and soil factors, the data with the case number of 75 were determined to be outliers and thus were removed from the dataset; in the analysis between BM and soil factors, the data with case numbers of 75 and 86 were determined to be outliers and were removed. No outliers were found in the multiple regression analysis between RIC and soil factors or in that between DIV and soil factors.

Supplementary Information S4. Procedures of multiple regression modeling

The process of multiple regression modeling is described as follows: firstly, variables with the largest effects on the dependent variable are entered into the model; then, the variable inside the model with the smallest partial regression sum of squares is selected and an F test is performed; if the test is significant, then all variables are kept in the model; if the test is not significant, this variable is removed from the model; then an F test for the other variables is performed in the same way until the F test of the variables in the model is significant; finally, the variable not included in the model with the largest partial regression sum of squares is selected an F test is performed; if the test is significant, then this variable is entered into the model; after the new variable is entered into the model, the variables inside the model undergo another round of F test to confirm that all their effects on the dependent variable are still significant; the variables outside of the model are selected again until all of their F test are not significant; the variables are entered into or removed from the model in this way until no variable in the model can be removed from it and no variable outside the model can be entered into it.

Supplementary Information S5. Test of linear relationship, data normality, and outliers before the multiple regression analyses between vegetation recovery and cycles of soil carbon and nutrients

Prior to the multiple regression analyses between vegetation recovery (parameters and general index) and cycles of soil carbon and nutrients, we calculated the Pearson correlations between them and found that COV, BM, RIC, DIV and REC were all significantly correlated with indices of C, N, P and K cycling. All indices of C, N, P and K cycling were thus involved in each multiple regression analysis for COV, BM, RIC, DIV and REC. All indices of C, N, P and K cycling and REC were tested for normality and found to be normally distributed. The outliers were also determined and removed before the OLS modeling. In the multiple regression analysis between COV and cycles of soil C, N, P and K, the data of case number 75 were found to be outliers and were removed; in that between BM and cycles of soil C, N, P and K, the data of case number 86 were found to be outliers and were removed.

Supplementary Information S6. Test of assumptions for multiple regression analyses

First, we test the assumptions of the multiple regression analyses between vegetation recovery and individual soil factors.

(1) Measurement level

It is assumed that dependent variables are measured at a level of interval or higher [5]. The dependent variables of COV, BM, RIC and DIV were all measured at the level of interval and thus fulfilled the assumption of measurement level for multiple regression analysis.

(2) Normality of dependent variables

The dependent variable (criterion) in a regression model is assumed to be approximately normally distributed [5]. For this assumption, we used the quotients of Skew/SES (skewness/standard error of skewness) and Kurt/SEK (kurtosis/standard error of kurtosis), to test the normality of the dependent variables. For all of the four criteria of COV, BM, RIC and DIV, both quotients of Skew/SES and Kurt/SEK were within the normal range of ±1.96 (Supplementary Table S2), indicating a normal distribution of the data [5,6].

(3) Linearity

Under this assumption, a linear relationship between each predictor and the criterion in a specific model is assumed [5,7]. We calculated Pearson's correlation coefficients between each

dependent variable and each soil factor and put only those factors significantly correlated with the dependent variable into the multiple regression analyses. There were therefore linear relationships between each parameter of vegetation recovery and the corresponding soil factors in the regression analyses.

(4) Normality of residuals

The errors (residuals) are assumed to be normally distributed [5,7,8]. This assumption was tested by examining the histogram and normal "probability-probability" plot (P-P plot) of the standardized residuals. The histograms of the residuals for all four criteria including COV, BM, RIC and DIV clearly showed that the distributions were normal (Supplementary Fig. S1). Moreover, most points were around the straight diagonal line with minor deviations in the normal P-P plot for the residuals of all four criteria (Supplementary Fig. S2). We therefore conclude that the shapes of distributions for the residuals of all four dependent variables analyzed in this study approached the shape of a normal curve.

(5) Homoscedasticity

The variances of prediction errors are assumed to be approximately constant for all predicted values of the criterion [9]. This is an assumption of homoscedasticity [8]. This assumption was tested by examining the scatterplot of the standardized residuals against the standardized predicted values. In each scatterplot, the points were distributed equally along the horizontal line for each

level of the standardized predicted value (Supplementary Fig. S3), suggesting that the data are homoscedastic [5].

(6) Multicollinearity

Under this assumption, it is assumed that all predicators in a specific regression model are not strongly correlated with one another [7]. A violation of this assumption leads to a multicollinearity between the predicators. An extreme multicollinearity results in problems such as error estimation [10] and the separation of the influences of the highly interrelated predicators [11], although a linear correlation between two predictors in a model is usually not serious enough to impact the regression analysis and, in practice, correlations between predictors are very common [7]. This assumption is normally tested by measuring the statistical quantity of variance inflation factor (VIF) and the tolerance. It is widely accepted that VIF > 10 and tolerance < 0.1 suggest a serious multicollinearity [7,10,11]. In the case of our study, the maximum value of VIF in all four regression models was much less than 10 and the minimum value of tolerance was greater than 0.1 (Supplementary Table S3), indicating no multicollinearity problems in our multiple regression analyses.

The assumptions of multiple regression analyses between vegetation recovery and cycles of soil C, N, P and K were tested in the same way described above. To avoid going into too much detail, the description of the assumption tests in this part is not shown, as most of the analysis is

similar to that shown above. Nevertheless, we tested all the assumptions and found all of them to be fulfilled.

Based on the above analysis, we conclude that the data gathered in this study meet all the assumptions. It is therefore reasonable to conduct the multiple regression analysis. Models built in the multiple regression analyses are therefore completed on the basis of assumption fulfillment.

References

5 Abu-Bader, S. H. *Advanced & Multivariate Statistical Methods for Social Science Research*. (Lyceum Books, 2010).

6 Abu-Bader, S. H. *Using Statistical Analysis in Social Work Practice: A Complete SPSS Guide*. (Lyceum Books, 2006).

7 Chatterjee, S. & Hadi, A. *Regression Analysis by Example*. 4 edn (Wiley, 2006).

8 Chatterjee, S. & Simonoff, J. S. *Handbook of Regression Analysis* (Wiley, 2013).

9 Tabachnick, B. G. & Fidell, L. S. *Using Multivariate Statistics*. 2 edn (Harper, 1989).

10 Mendenhall, W. & Sincich, T. *A Second Course in Statistics: Regression Analysis*. 7 edn (Pearson Education, 2012).

11 Rawlings, J. O., Pantula, S. G. & Dickey, D. A. *Applied Regression Analysis: A Research Tool*. 2 edn (Springer Texts in Statistics, Springer, 1998).

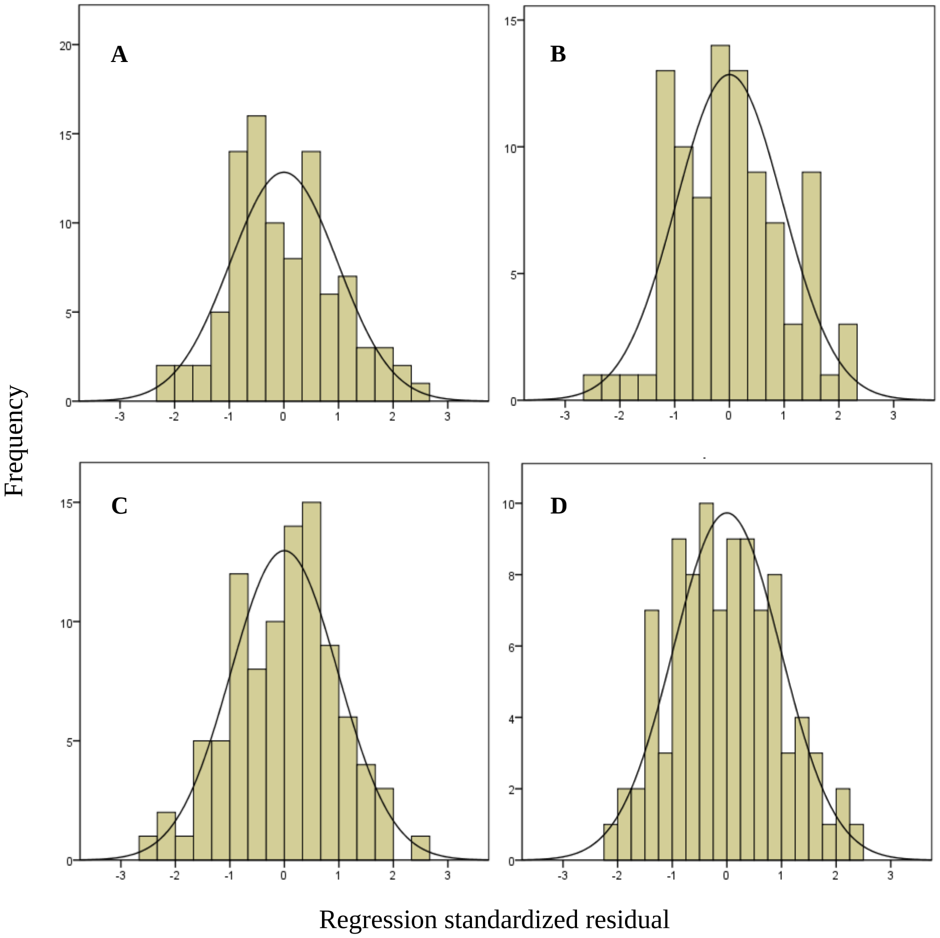

Supplementary Fig. S1 Histogram and normal curve for the residuals of plant cover (A), plant biomass (square-root transformed) (B), plant species richness (C) and plant species diversity (D).

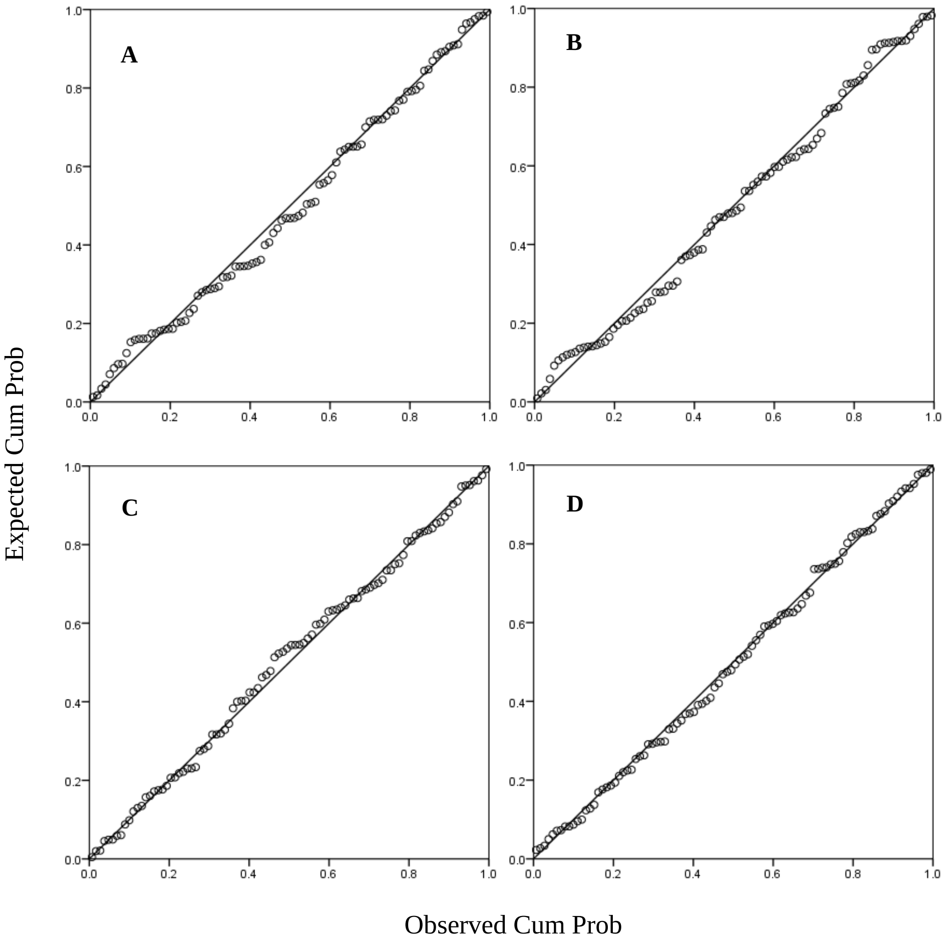

Supplementary Fig. S2 Normal P-P plot for the residuals of plant cover (A), plant biomass (square-root transformed) (B), plant species richness (C) and plant species diversity (D). Expected Cum Prob: expected cumulative probability; Observed Cum Prob: observed cumulative probability.

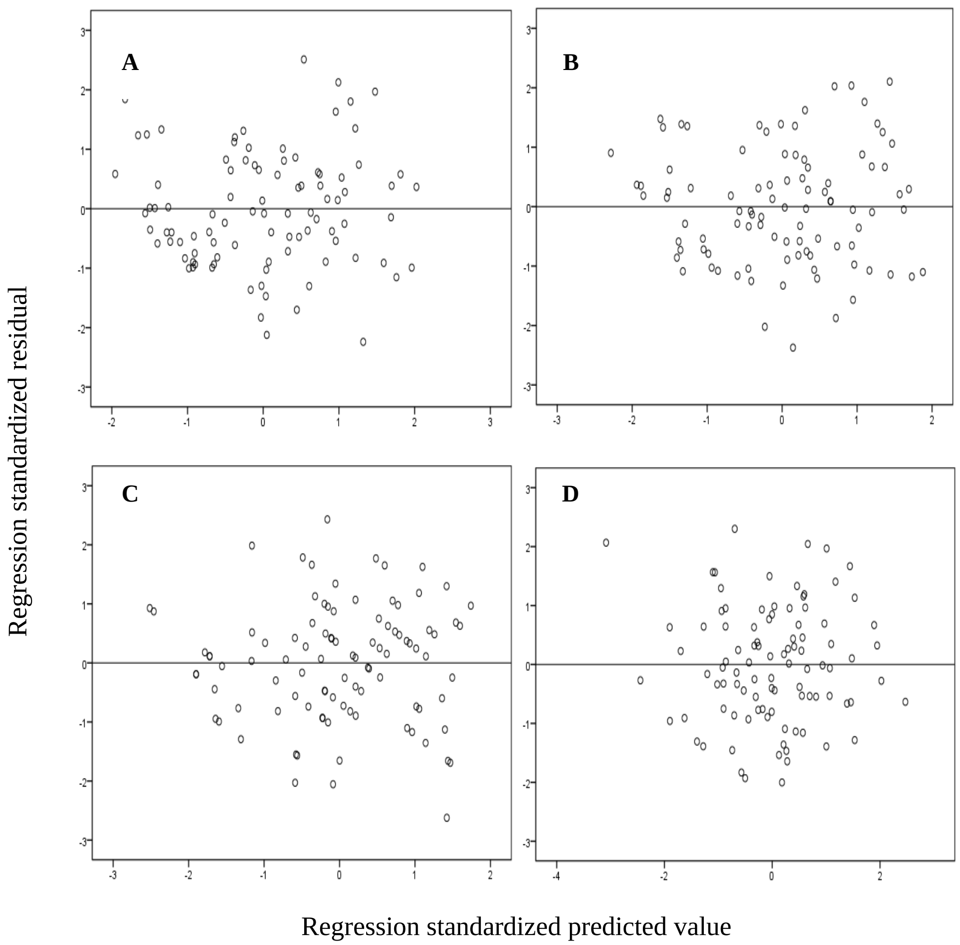

Supplementary Fig. S3 Scatterplot for the residuals of plant cover (A), plant biomass (square-root transformed) (B), plant species richness (C) and plant species diversity (D), against their respective predicted values.

Supplementary Table S2 Skewness, kurtosis and quotients with their standard errors of plant cover, plant biomass, plant species richness and plant species diversity

	Skew	SES	Skew/SES	Kurt	SEK	Kurt/SEK
Cover	0.340	0.247	1.377	-0.788	0.490	-1.608
Biomass (Sqrt trans)	0.278	0.249	1.116	-0.659	0.493	-1.337
Richness	-0.024	0.246	-0.098	-0.830	0.488	-1.701
Diversity	0.070	0.246	0.285	-0.467	0.488	-0.957

Skew: skewness; SES: standard error of the skewness; Skew/SEM: the quotient of the skewness divided by its standard error; Kurt: kurtosis; SEK: standard error of the kurtosis; Kurt/SEK: the quotient of the kurtosis divided by its standard error; Sqrt transf: square-root transformed.

Supplementary Table S3 Collinearity statistics of multiple regression models for plant cover, plant biomass, plant species richness and plant species diversity

Dependent variable	Model	Collinearity Statistics	
		Tolerance	VIF
Cover	Constant		
	AK	0.823	1.215
	SOC	0.897	1.114
	CS	0.858	1.165
Biomass (Sqrt trans)	Constant		
	AK	0.575	1.740
	TN	0.325	3.076
	SOC	0.601	1.663
	PRO	0.456	2.191
	PHO	0.495	2.020
Richness	Constant		
	TN	0.255	3.922
	PHO	0.516	1.937
	CLS	0.301	3.317
Diversity	Constant		
	PRO	0.622	1.608
	SW	0.950	1.053
	AK	0.619	1.616

VIF: variance inflation factor; Sqrt trans: square-root transformed. AK: soil available potassium; SOC: soil organic carbon; CS: soil coarse sand fraction; TN: soil total nitrogen; PRO: soil protease activity; PHO: soil phosphatase activity; CLS: soil clay and silt fraction; SW: soil water content

Supplementary Information S7. Model fit indices, outliers, and multivariate normality of structural equation models

Several indices, such as the comparative fit index (CFI), the incremental fit index (IFI), the Chi-square (χ^2) and the standardized root mean square residual (SRMR) were utilized to evaluate the model fit (Supplementary Table S4). CFI is taken as an index of "goodness-of-fit" [12,13] and varies between 0 and 1 [12,14]. A higher CFI indicates a better fit between the model and the data [13]. The value of CFI or IFI close to 1 suggests a very good fit [15]. CFI, IFI and the normed fit index (NFI) are equivalent indices of comparative fit [14]. Both CIF and IFI show little variability to changes in sample size, whereas NFI is sample size dependent and may therefore show large bias in the estimation [14,16]. Therefore, in the present study we employed CIF and IFI but not NFI to evaluate the model fit. SRMR is an index of model fit evaluating the ability of the model to reproduce the sample data [12]. Simultaneously, it is an index for "badness-of-fit" and when its value is close to 0, this suggests a good model fit [13].

Prior to the structural equation modeling, the data were normalized in the same manner as before the OLS modeling. After the estimates calculated in AMOS, data of case numbers of 33 and 86 were found to be outliers for both SEM1 and SEM2, with the largest Mahalanobis distance and very small probabilities in the p2 column ($p < 0.05$) shown in the AMOS output. The data of all variables with case numbers of 33 and 86 were therefore removed from the data set and the estimates were then calculated again. Results showed no more outliers and a multivariate normal distribution with the c.r. values of the multivariate kurtosis of -0.42 and -0.35 for SEM1 and SEM2, respectively, which were both less than |1.96|.

Supplementary Table S4 Model fit indices of structural equation models shown in Figs. 1 and 2 of the main text

	SEM without latent variables	SEM with latent variables
n	96	96
Chi-square (χ^2)	41.10	147.85
df	24	40
CFI	0.98	0.86
IFI	0.98	0.86
SRMR	0.064	0.12

n: number of cases; df: degree of freedom; CFI: comparative fit index; IFI: incremental fit index; SRMR: standardized root mean residuals.

References

12 Hu, L. t. & Bentler, P. M. Cutoff criteria for fit indexes in covariance structure analysis: Conventional criteria versus new alternatives. *Struct. Equ. Modeling* **6**, 1-55, doi:10.1080/10705519909540118 (1999).

13 Hacker, N. *et al.* Plant diversity shapes microbe-rhizosphere effects on P mobilisation from organic matter in soil. *Ecol. Lett.* **18**, 1356-1365, doi:10.1111/ele.12530 (2015).

14 Bentler, P. M. Comparative fit indexes in structural models. *Psychol. Bull.* **107**, 238-246, doi: 10.1037/0033-2909.107.2.238 (1990).

15 Arbuckle, J. L. *IBM SPSS Amos 21 User's Guide* (IBM Corporation, Amos Development Corporation, 2012).

16 Bollen, K. A. A new incremental fit index for general structural equation models. *Sociol. Method Res.* **17**, 303-316, doi:10.1177/0049124189017003004 (1989).

Supplementary Information S8.

Supplementary Table S5 A collection of all abbreviations used in this paper and their corresponding full forms

Abbreviations	Corresponding full forms
AK	soil available potassium
AN	soil available nitrogen
AP	soil available phosphorus
B	unstandardized coefficient in a regression model
BD	soil bulk density
BEF	biodiversity-ecosystem functioning
Beta	standardized coefficient
BM	plant biomass
95% con inter	95% confidence interval for B
C	carbon
CAT	soil catalase activity
CFI	comparative fit index
CLS	soil clay and silt fraction

Abbreviations	Corresponding full forms
COV	plant cover
CS	soil coarse sand fraction
D	dry weight of the soil sample
df	degrees of freedom
DIV	plant species diversity
DRS	desertification reversal stage
EC	soil electrical conductivity
Expected Cum Prob	expected cumulative probability
FS	soil fine sand fraction
H′	Shannon - Wiener index
IFI	incremental fit index
INV	soil invertase activity
Kurt	kurtosis
N	nitrogen
NFI	normed fit index
NI	total number of individuals for all plant species

Abbreviations	Corresponding full forms
n	number of cases
n_i	number of individuals for species i
NC	ecosystem functions related to carbon and nutrient cycling
K	potassium
Observed Cum Prob	observed cumulative probability
OLS	ordinary least square
P	phosphorus
P_f	p value of the F-test for the overall significance of the model
P_i	proportion of the abundance of species i to that of all species
P_t	p value of the t-test for an individual regression coefficient
PHO	soil phosphatase activity
P-P plot	normal "probability-probability" plot
PRO	soil protease activity
REC	general index of vegetation recovery
RIC	plant species richness
SAP	soil abiotic properties

Abbreviations	Corresponding full forms
SEB	standard error of B
SEE	standard error of the estimate
SEM	structural equation modeling
SEM1	structural equation model without latent variables
SEM2	structural equation model with latent variables
SEK	standard error of kurtosis
SES	standard error of skewness
Skew	skewness
SOC	soil organic carbon
Soil Facs	soil factors
Sqrt trans	square-root transformed
SRMR	standardized root mean square residual
SW	soil water content
TK	soil total potassium
TN	soil total nitrogen
TP	soil total phosphorus

Abbreviations	Corresponding full forms
URE	soil urease activity
V	volume of the cutting ring
Vege Recov	parameters of vegetation recovery
VFS	soil very fine sand fraction
VIF	variance inflation factor
W	wet weight of the soil sample
χ^2	chi-square
